# Prediction of the Proximal Humerus Morphology Based on a Statistical Shape Model with Two Parameters: Comparison to Contralateral Registration Method

**DOI:** 10.3390/bioengineering10101185

**Published:** 2023-10-13

**Authors:** Florianne E. van Schaardenburgh, H. Chien Nguyen, Joëll Magré, Koen Willemsen, Bert van Rietbergen, Stefaan Nijs

**Affiliations:** 1 Orthopaedic Biomechanics, Department of Biomedical Engineering, Eindhoven University of Technology, 5612 AZ Eindhoven, The Netherlands; 2 Department of Orthopaedics, University Medical Center Utrecht, 3584 CX Utrecht, The Netherlands; 3 3D Lab, University Medical Center Utrecht, 3584 CX Utrecht, The Netherlands; 4 Division Surgical Specialties, Department Trauma Surgery, University Medical Center Utrecht, 3584 CX Utrecht, The Netherlands

**Keywords:** statistical shape modelling, proximal humerus fractures, humeral anatomy, 3D planning

## Abstract

(1) Background: Complex proximal humerus fractures often result in complications following surgical treatment. A better understanding of the full 3D displacement would provide insight into the fracture morphology. Repositioning of fracture elements is often conducted by using the contralateral side as a reconstruction template. However, this requires healthy contralateral anatomy. The purpose of this study was to create a Statistical Shape Model (SSM) and compare its effectiveness to the contralateral registration method for the prediction of the humeral proximal segment; (2) Methods: An SSM was created from 137 healthy humeri. A prediction for the proximal segment of the left humeri from eight healthy patients was made by combining the SSM with parameters. The predicted proximal segment was compared to the left proximal segment of the patients. Their left humerus was also compared to the contralateral (right) humerus; (3) Results: Eight modes explained 95% of the variation. Most deviations of the SSM prediction and the contralateral registration method were below the clinically relevant 2 mm distance threshold.; (4) Conclusions: An SSM combined with parameters is a suitable method to predict the proximal humeral segment when the contralateral CT scan is unavailable or the contralateral humerus is unhealthy, provided that the fracture pattern allows measurements of these parameters.

## 1. Introduction

Proximal humerus fractures (PHFs) account for approximately 6% of all human fractures [1]. Adequate treatment of PHFs has gained importance due to a predicted increase in incidence and fracture complexity as life expectancy globally increases, resulting in tremendous healthcare burdens [2]. At the moment, at least 21% of all PHFs are difficult to treat 3- and 4-part fractures [1]. Four-part PHFs are often composed of the lesser tuberosity, greater tuberosity, humeral head, and shaft. After trauma, the rotator cuff insertions and capsular attachments may exert tension on the fragments, resulting in fragment displacement [3]. Approximately half (51%) of all PHFs are displaced, with the majority (77%) involving the surgical neck [4]. In addition to displacement, impaction can result in volumetric bone loss in the toroidal region between the head and the shaft, which is an important functional and anatomical area [5,6]. Both displacement and volumetric bone loss make PHFs more difficult to treat conservatively.

Currently, there is an increasing tendency towards a surgical approach for treating complex 3- and 4-part PHFs, as it has been shown to yield improved outcomes compared to conservative treatments [2,7,8]. However, impaction and displacement present surgical challenges due to the altered morphology; 30% of these surgically treated complex PHFs are accompanied by complications such as infections, avascular necrosis, non-union or malunion, and implant-related failures [9,10,11,12]. 

During surgery, the surgeon’s view of the humerus is limited due to the rotator cuff, and surrounding vital structures, such as blood vessels. Accurate visualization of the fragments is essential for a successful outcome, yet it is difficult during surgery. Therefore, a computed tomography (CT) scan and/or an X-ray of the fractured humerus is made. However, two-dimensional (2D) images may not always be sufficient for a thorough understanding of the fracture, as they offer only a single perspective of the fractured humerus, making it difficult to assess the depth and extent of the displacement and may result in inadequate preoperative planning [13,14]. 

The golden standard for determining the premorbid anatomy of a fractured bone is to mirror the contralateral side and use this as a template for reconstruction. However, this approach has limitations; it necessitates a CT scan of both arms whereof the contralateral humerus needs to be healthy and there may be a variance between the dominant and non-dominant humeral morphology [15,16]. This causes the approach to be not optimal.

A better understanding of the full three-dimensional (3D) displacement would provide additional insight into the fracture morphology and bone impaction, which is important for the presurgical planning of an anatomical reduction. A complete anatomical reduction is key to a successful outcome after surgical treatment [10]. A difference in the position and size of the articular surface by 4–5 mm alters the kinematics and forces across the glenohumeral joint, leading to unacceptable outcomes [17,18,19]. Even displacements as small as 3 mm of a tuberosity fragment can negatively influence the outcomes in active patients. Defining the premorbid morphology before surgery also provides insight into fixation strategies. One promising method is to use a Statistical Shape Model (SSM) to predict the complete premorbid morphology of the humerus [20]. SSM is a technique that facilitates the grouping and characterization of shape variation into a set of components that allow for a quantitative description of shape morphologies [21]. In many areas of biology and medicine, the SSM has proven useful as a new generation of approaches for the analysis of anatomical shapes [22].

This study aimed to create an SSM of the humerus for predicting the morphology of the humeral proximal segment with a precision of 2 mm or better, offering an alternative to the contralateral registration method. To do so, we first developed an SSM model of the humerus based on two data sets. Following we defined three geometric parameters that can be easily measured from a 3D model of a CT scan of a humerus and investigated to what accuracy the selection of a model from the SSM based on these parameters can describe bone shape. Finally, we compared the shape derived from this SSM approach with the traditional approach based on the contralateral side.

## 2. Materials and Methods

### 2.1. Data

**Figure 1:**
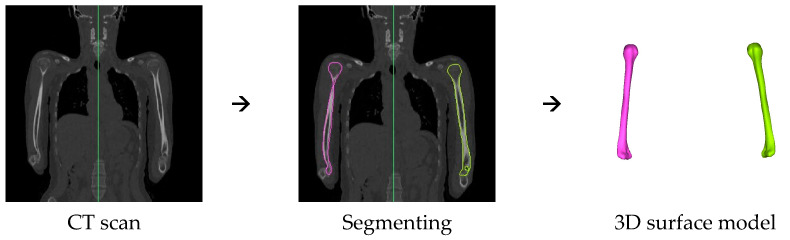
The steps from CT scan to 3D model.

Two datasets were available to construct the SSM. The first was a dataset of 150 anonymized full-body CT scans from the University Hospital Leuven, Belgium, with an average age of 43.9 years (SD 16.5 years) and the second was a database of 28 CT scans from the University Medical Center Utrecht, The Netherlands (IRB Protocol Number 16-612/C), with an average age of 28.2 years (SD 4.7 years). All healthy and complete humeri were included, resulting in a total of 137 humeri. This set comprised the humeri of 41 men and 41 women. The database originating from Leuven had been previously utilized in a study by Dauwe et al. [23]. For validation of the SSM, a third dataset was used with eight full-body CT scans. This dataset was selected from the New Mexico Decedent Image Database [24]. This dataset was chosen to only include healthy and complete humeri from females and males at the ages of 30, 40, 60, and 70 years, which resulted in one male and one female from each age category. All humeri were segmented in the software Materialise Mimics (25.0, Materialise NV, Leuven, Belgium) and converted into 3D surface mesh models (STL files), with a mean triangular edge length of 1.5 mm (Figure 1). The meshes were smoothed in Materialise 3-Matic (17.0, Materialise NV, Leuven, Belgium), and all right humeri were mirrored to the left.

### 2.2. SSM Creation

**Figure 2:**
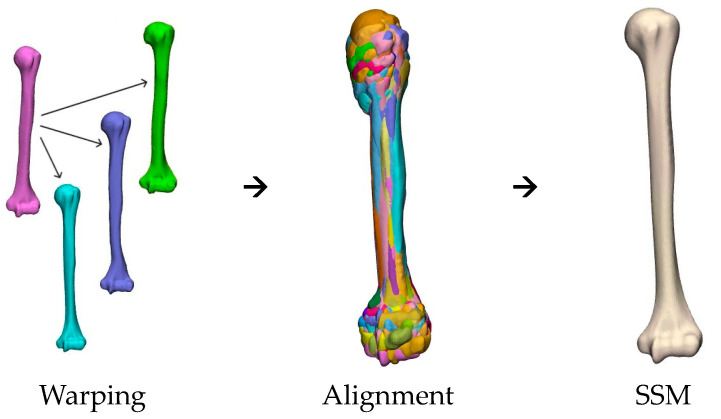
The steps for creating the SSM.

The SSM was generated in three subsequent steps using the software 3-Matic, as visualized in Figure 2. First, one random humerus from the dataset called the reference humerus, was warped to all other humeri one by one [25]. All humeri needed to be made up from the same mesh to ensure that all humeri in the dataset had an equal number of points. The warped humeri and the reference humerus were used to create the SSM. 

Second, to ensure correct alignment correspondence, all humeri were manually aligned first, followed by the application of the iterative closest point (ICP) method between all humeri [26]. This alignment process eliminated rotational and translational variations [20,27]. To keep the size variation of the humeri, no scaling was performed.

Third, principal component analysis (PCA) was used to describe the mean shape, the principal components (PCs), also known as modes of variation, and their weighting factors. Shape variations were modeled using a normal distribution: S~N(μ,∑). The mean shape (*μ*) provided information about the general shape of the database, and the variation (*∑*) provided information about the differences between patients. A random humerus was, therefore, described by the formula: $S=\mu +\sum_{i=1}^{n}w_{i}m_{i}$, where *n* was the number of shapes included in the SSM, *w_i_* was the weighting factor, and *m_i_* was the mode of variation. To assess the model’s quality, compactness was evaluated, which encoded the percentage of variance captured by a specific number of PCs, indicating how efficiently the shape variation in the dataset was represented by the SSM. For a detailed explanation of the parameter description of the principal components, a reference is made to the study by Dauwe et al. [23].

### 2.3. Definition of Geometric Parameters

To create reproducible measurements on the humeri, a humeral coordinate system (HCS) was defined following the recommendations from the International Society of Biomechanics on definitions of joint coordinate systems [28]. The origin of the HCS was defined as the center of a sphere fitted to the articular surface of the proximal humerus. The *Y*-axis was defined as a line through the center of the humeral head and the midpoint between the two distal epicondyles. The *x*-axis was a line perpendicular to the plane through the origin, and both caudal points on the epicondyles. The *Z*-axis was perpendicular to both the *Y*- and *X*-axis. 

**Figure 3:**
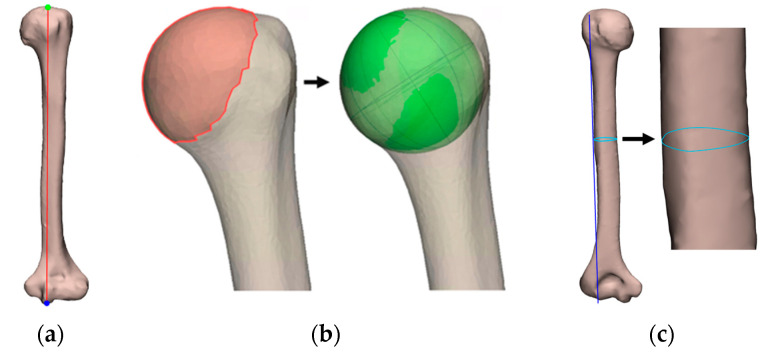
Illustration of the parameters measured on a humerus: (**a**) The maximum humerus length; (**b**) The humeral head radius; (**c**) The shaft circumference.

The parameters below were identified on the 3D models and based on literature and used to predict the humeral morphology (Figure 3) [20,23,29,30,31,32,33,34,35]:

1. The maximum humerus length was defined as the direct distance from the most superior point of the humeral head to the most inferior point of the trochlea. Two planes were created normal to the *Y*-axis. One plane was translated proximally until it intersected with the most superior point of the proximal humerus, and one plane was translated distally until it intersected with the most superior point of the distal humerus. The direct distance between the planes served as the maximum humerus length;
2. The humeral head radius was defined as the radius of the fitted sphere into the humeral head.
3. The shaft circumference was defined as the circumference around halfway the length of the diaphysis. A plane was created normal to the *Z*-axis. This plane was moved towards the diaphysis until it intersected halfway along the length of the diaphysis. This was the most protruding point on the deltoid tuberosity. At this height, the circumference of the shaft was measured by creating a plane normal to the *Y*-axis and cutting the humerus.

### 2.4. Testing the Combinations of Parameters

The three parameters were measured on five random humeri from the SSM database. All measurements were repeated by the same observer after two months to calculate the intra-observer reliability as quantified by the intraclass correlation coefficient (ICC). 

Measurements were added to each vector of N vertices × 3 coordinates (which represented one patient in the training set) using the Materialise Advanced SSM Viewer plug-in (17.0, Materialise NV, Leuven, Belgium). The plugin manual stated that “the most important addition provided by the plugin is the ability to control the SSM through measurements (instead of nodes)”. The principal component was recalculated, now including the measurements. Since the measurements were then part of the SSM, it was possible to set a target value for the measurements and the modes of the SSM were updated accordingly [36].

Depending on fracture type, not all three parameters may be accurately measurable in the fractured case (in particular length). Also, some of the selected parameters may be better predictors for the surface geometry than others. For this reason, a humerus prediction was made for different combinations of the tree parameters (seven cases per humerus). The most optimal combination of parameters, providing the most accurate prediction, was used as the predictive parameters. 

### 2.5. Original Humerus vs. Predicted SSM Humerus

For the comparison of the original left humerus versus the predicted humerus, first, the predictive parameters were measured on all left humeri in the validation database (N = 8). These values were set as a target value in the plugin, after which a predicted humerus was obtained. For comparison, the original and predicted humeri were aligned in the same manner as described in step two of the SSM creation. Second, they were cut perpendicular to the axis of the humeral diaphysis cylinder at 6 cm from the most proximal point. The upper part was called the proximal segment. Warping was performed to obtain an equal number of points in the mesh of the proximal segments for all humeri (1952 points). Third, a part comparison between the two segments was conducted. This provided the deviation in mm between the two humeri for every point on the proximal segment. The deviation was categorized as 0–1 mm, 1–2 mm, and >2 mm. In our research, we have chosen to use 2 mm as the clinically relevant threshold because displacements larger than this threshold may influence the clinical outcomes. Results were quantified as the % of the proximal segment surface for which the deviation exceeded these thresholds. 

### 2.6. Original Humerus vs. Contralateral Humerus

For the comparison of the original humerus versus the contralateral humerus, all right humeri (N = 8) from the validation database were mirrored to the left. Next, the two humeri were cut, warped, and compared as described in the previous paragraph. This process was repeated for all eight sets of left and right humeri in the validation database. 

## 3. Results

### 3.1. Statistical Shape Model

**Figure 4:**
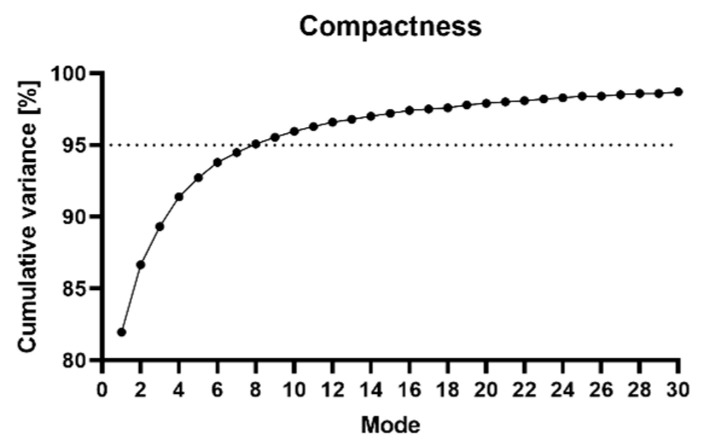
The compactness of the SSM. Eight modes explain 95% of the variation.

**Figure 5:**
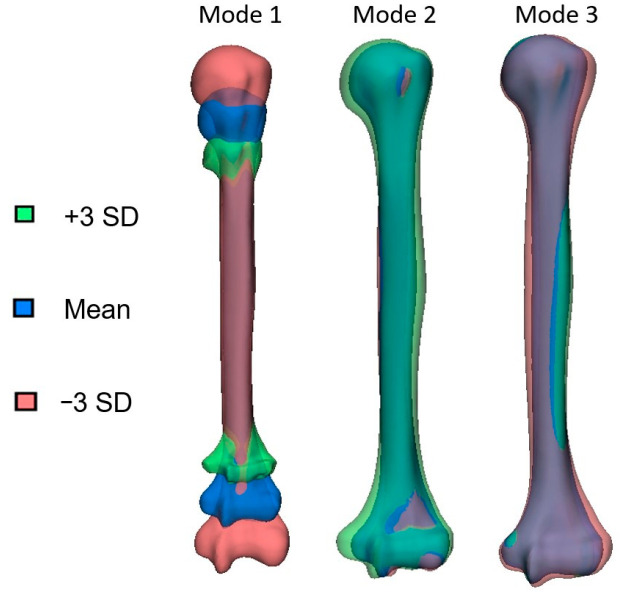
The first three modes of variation of the SSM of the humerus. Variation in the shape can be visualised by changing the standard deviation (SD), which ranges from −3SD (red coloured) to +3SD (green coloured).

The SSM was built using 137 healthy humeri. The compactness of the SSM revealed that eight modes explained 95% of the variation, as shown in Figure 4. Figure 5 visualizes the first three modes of variation of the SSM. The mean, −3, and +3 standard deviations are depicted and colored in the same image. Dauwe et al. [23] showed that the first principal component displayed the most prominent variation, which was the humeral length. The second principal component accounted for variations in the head inclination angle, medial head offset, retroversion angle, and lesser tuberosity offset. 

### 3.2. Parameters

The ICC for the repeated measurements was found to be 0.99, indicating excellent agreement between both measurements.

Table 1 summarizes the accuracy of the seven predictive parameter combinations that were measured on five random humeri. The combination of length and radius resulted in the lowest percentage of the proximal segment surface area with a deviation >2 mm and the lowest maximum deviation, compared to the other parameter combinations. Therefore, this combination was used for predicting the humeral morphology of the validation set.

### 3.3. SSM Prediction vs. Contralateral Registration Method

**Figure 6:**
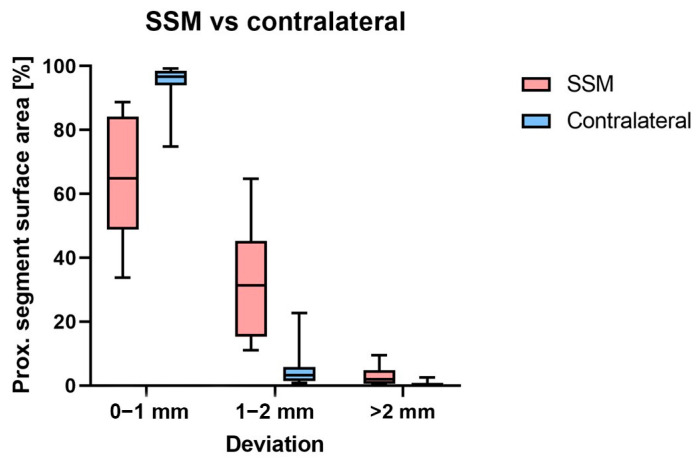
The proximal segment of the SSM prediction and the contralateral registration method have different deviations. The boxplots show the minimum and maximum values.

The percentages of the proximal segment surface area with a deviation between 0 and 1 mm, 1 and 2 mm, and >2 mm of the SSM prediction and the contralateral registration method, can be seen in Table 2 and Figure 6. 

## 4. Discussion

This study aimed to create a humeral Statistical Shape Model that would allow for the prediction of the proximal segment of the humerus. This was achieved by creating an SSM from 137 humeri and defining three parameters (humerus length, head radius, and circumference) as the basis for this model prediction from the SSM. The primary findings of this study were that most deviations of the SSM prediction and the contralateral registration method were below the clinically relevant 2 mm distance threshold. 

Humeral SSMs are gaining popularity due to their use in the rapid generation of patient-specific bone models [37]. The major benefit of using an SSM for predicting the premorbid anatomy over the golden standard, i.e., the contralateral registration method, is that it can be used even when trauma has happened to both arms or a pathology is present. Additionally, there are differences between dominant and non-dominant arms. There is more humeral retrotorsion in the dominant arm of almost all overhead-throwing athletes, irrespective of their gender [16,38,39]. Although these differences do not alter the proximal morphology, they demonstrate that the contralateral side may not be a suitable template for every patient. 

Literature suggests that an alteration in the positioning of the proximal humeral surface of 4 to 5 mm can result in abnormal glenohumeral biomechanics [17,18]. Bono et al. [40] tested eight fresh human cadaver shoulders in a dynamic testing apparatus and found that the abduction force was significantly increased by 16% by superior displacements of 0.5 cm. Small amounts of displacement may alter the balance of forces. Our study shows that, on average, 2.9% (SD 3.1%) of the proximal segment surface area of the predicted humerus had a deviation of more than 2 mm from the original humerus. A predicted humerus with a surface area this small does not necessarily lead to incorrect reduction of the fracture site as fragments are reduced based on the mean displacement of the complete fragment. The greater tuberosity typically measures an average width of 31.39 ± 2.74 mm and an average height of 27.11 ± 2.57 mm [41]. Therefore, deviations above 2 mm that only occur in 2.9% of the surface may not be clinically relevant. Subsequently, the use of a humeral SSM is an acceptable alternative when the scan of the contralateral side is unavailable or otherwise affected.

Potential differences in the populations contributing to the SSM and validation datasets may have influenced the results. The SSM database included only Dutch and Belgian patients, whereas the validation database was obtained from American patients. Goldberg et al. [42] discovered that specimens from Caucasian cadavers showed greater retroversion and more valgus neck-shaft angle than specimens from Afro-American cadavers. In our study, the part comparison between the predicted SSM humeri and the original humeri showed a positive deviation, i.e., an overestimation, in 76% of proximal segment surface areas. This demonstrates that most of the time the reconstructed humerus was wider compared to the original. This might be explained by the differences in humeral morphology between the SSM database and the validation database. The Dutch and Belgian populations might be taller than the Americans, and this could be reflected in their humeri. Nevertheless, the validation set is small and could include both Mexican immigrants and Caucasians of European descent. 

It should be kept in mind that the measurements of maximum humerus length, humeral head radius and shaft circumference cannot always be measured on the humerus, depending on the fracture pattern. In 4-part PHFs, the humeral head is often dislocated, restricting possibilities for measuring the total length of the humerus. Our research has assumed that the length is still measurable after the fracture. It is expected that the humeral head radius is measurable as this parameter only depends on the articular surface, and this is often intact after a fracture [43].

There were limitations related to this study. First, all measurements conducted on the humeri were conducted manually, but given the excellent ICC, it is not expected that this will influence the results much. Second, our study did not investigate the neck-shaft angle, while this is an important anatomical variable defining the humerus. For this, the angle between the humeral proximal articular surface and the intramedullary axis of the humeral shaft could have been measured. We were only interested in proximal reduction and did not investigate any angles. Therefore, our SSM could not be used to predict the neck-shaft angle. Although the second PC accounted for other shape variations, it was chosen to not include these as parameters to keep the measurements limited. 

Based on our work, one could suggest extending the SSM database with humeri from various populations, ages, and sexes to capture the widest range of shape variations. In the current study, the included patients were all adults. It would be insightful to extend the predictive power of our model with adolescents, by including them into the SSM. Of note, proximal humerus fractures, by means of complex fractures in need of surgical reduction, are predominately present in older patients. Next to the inclusion of adolescents, the SSM predictive accuracy is possibly enhanced when the database is subdivided into specific age ranges or genders, whereafter the humeral morphology of a PHF can be predicted based on data matched to the patient’s age and gender. This would be interesting to research in future studies. Also, in addition to using the deviation of the complete surface area as an evaluation method, the deviation of specific landmarks could be measured. This could provide information about the humeral characteristics, such as the neck-shaft angle or humeral head height [23,35]. To overcome the restriction that the maximum humerus length is not always measurable, a prediction of the proximal segment could be conducted by using the distal segment knowledge as this is intact with a PHF. The proximal segment could be predicted by using the provided partial data to fit the SSM. This could be conducted by fitting one humerus into another using anatomical landmarks. A computer algorithm could compute the number, location, and precision of the landmarks [15]. Treatment of PHFs often involves repositioning of bone fragments after which osteosynthesis is performed with plate fixation [44]. Open reduction with internal fixation has the potential to yield favorable functional outcomes; however, orthopedic trauma surgeons are concerned about the depth and extent of the displacement of bone fragments caused by cancellous bone compression, complicating the treatment and likely to increase the failure rate. Proper reduction and filling of bone defects is essential for good clinical outcomes [45]. In the future and based on the current performed study, the predicted proximal segment of the humerus could be used as a template for the reduction of the fragments pre-surgically, enhancing the understanding of PHF fracture displacement and bone voids beforehand. The 3D insights into these PHF fracture parameters are then to be used for quantitative measurements, providing valuable metrics for fracture complexity. Finally, this may lead to a new classification system in which PHFs are classified based on the fragment displacements and bone voids in 3D. Also, the SSM could be combined with a finite element analysis to optimize virtual surgical planning. The finite element analysis may provide information about the optimal screw orientation in the predicted morphology. This improves fixation stability in PHFs and reduces mechanical failures [46]. Moreover, the humeral SSM model could be used for calculating regional defects after fracture reduction. These regional defects may give insight into the maximum threshold of the defect where osteosynthesis does not result in a good outcome and bone grafting may be necessary. 

## 5. Conclusions

In this study, a humeral SSM was built and used to predict the proximal segment of a humerus. The SSM prediction and the contralateral registration method both give clinically relevant information as they provide templates with predominantly deviations below 2 mm. It can be concluded that the use of an SSM with the parameters maximum humerus length and humeral head radius is a suitable method to predict the humeral proximal segment when the contralateral CT scan is unavailable, provided that the fracture pattern allows measurements of these parameters.

## Figures and Tables

**Table 1:** Part comparison of the seven different parameter combinations.

Parameter	Proximal Segment Surface Area with a Deviation > 2 mm	Maximum Deviation in the Proximal Segment Surface Area
Length	13.4% (SD 22.2%)	3.2 mm (SD 1.3%)
Radius	3.9% (SD 3.9%)	3.0 mm (SD 0.6%)
Circumference	23.0% (SD 17.9%)	3.6 mm (SD 0.8%)
Length + radius	2.8% (SD 3.6%)	2.8 mm (SD 0.6%)
Length + circumference	14.0% (SD 18.4%)	3.3 mm (SD 0.7%)
Radius + circumference	4.6% (SD 5.2%)	3.1 mm (SD 0.7%)
Length + radius + circumference	6.8% (SD 4.1%)	3.3 mm (SD 0.9%)

**Table 2:** Part comparison between SSM prediction and contralateral registration method; 100% is the total proximal segment surface area.

Deviation between Two Humeri	Percentage of the Proximal Segment Surface Area	
	Original Humerus vs. Predicted SSM Humerus	Original Humerus vs. Contralateral Humerus
0–1 mm	64.6% (SD 19.8%)	94.1% (SD 8.0%)
1–2 mm	32.5% (SD 18.4%)	5.5% (SD 7.2%)
>2 mm	2.9% (SD 3.1%)	0.4% (SD 0.9%)

## Data Availability

Data sharing not applicable.
